# A *Salmonella* Regulator Modulates Intestinal Colonization and Use of Phosphonoacetic Acid

**DOI:** 10.3389/fcimb.2017.00069

**Published:** 2017-03-15

**Authors:** Johanna R. Elfenbein, Leigh A. Knodler, Allison R. Schaeffer, Franziska Faber, Andreas J. Bäumler, Helene L. Andrews-Polymenis

**Affiliations:** ^1^Department of Microbial Pathogenesis and Immunology, College of Medicine, Texas A&M University Health Science CenterBryan, TX, USA; ^2^Department of Clinical Sciences, College of Veterinary Medicine, North Carolina State UniversityRaleigh, NC, USA; ^3^Paul G. Allen School for Global Animal Health, College of Veterinary Medicine, Washington State UniversityPullman, WA, USA; ^4^Department of Medial Microbiology and Immunology, School of Medicine, University of California DavisDavis, CA, USA

**Keywords:** *Salmonella*, phosphonates, mice, infection, phosphonoacetic acid

## Abstract

Many microorganisms produce phosphonates, molecules characterized by stable carbon-phosphorus bonds that store phosphorus or act as antimicrobials. The role of phosphonates in the marine biosphere is well characterized but the role of these molecules in the intestine is poorly understood. *Salmonella enterica* uses its virulence factors to influence the host immune response to compete with the host and normal microflora for nutrients. *Salmonella* cannot produce phosphonates but encodes the enzymes to use them suggesting that it is exposed to phosphonates during its life cycle. The role of phosphonates during enteric salmonellosis is unexplored. We have previously shown that *STM3602*, encoding a putative regulator of phosphonate metabolism, is needed for colonization in calves. Here, we report that the necessity of *STM3602* in colonization of the murine intestine results from multiple factors. *STM3602* is needed for full activation of the type-3 secretion system-1 and for optimal invasion of epithelial cells. The Δ*STM3602* mutant grows poorly in phosphonoacetic acid (PA) as the sole phosphorus source, but can use 2-aminoethylphosphonate. PhnA, an enzyme required for PA breakdown, is not controlled by STM3602 suggesting an additional mechanism for utilization of PA in *S*. Typhimurium. Finally, the requirement of *STM3602* for intestinal colonization differs depending on the composition of the microflora. Our data suggest that *STM3602* has multiple regulatory targets that are necessary for survival within the microbial community in the intestine. Determination of the members of the *STM3602* regulon may illuminate new pathways needed for colonization of the host.

## Introduction

In the intestine the microflora and the host vie for nutrients that vary in availability along the length of the intestine. *Salmonella* Typhimurium (STm) uses multiple strategies to gain nutrients and survive in this niche. Non-typhoidal *Salmonella*, including STm, secrete effectors via the type 3-secretion system-1 (TTSS-1). The effectors SipA, SopB, SopD, SopA, and SopE2 promote invasion, an influx of inflammatory cells, predominantly neutrophils, and alter the composition of the microbial flora (Zhang et al., 2002; Raffatellu et al., 2005). STm uses the tetrathionate and nitrate, produced in the intestine as a result of the inflammatory response, as terminal electron acceptors (Winter et al., 2010; Lopez et al., 2012). In addition, STm uses host-derived sugars, such as ethanolamine released by microbial damage in the intestine, as energy sources during inflammation (Thiennimitr et al., 2011). Metal scavenging through salmochelin and high-affinity transporters allow STm to acquire essential nutrients during infection (Raffatellu et al., 2009; Liu et al., 2012). While these are some essential mechanisms salmonellae use to acquire nutrients and replicate in the intestine, these mechanisms may represent only a small fraction of the metabolic potential of STm in the intestine.

Phosphonates are molecules with stable carbon-phosphorus bonds. Microorganisms produce these molecules to store phosphorus during periods of phosphate limitation (Villarreal-Chiu et al., 2012). In addition to a metabolic utility of these molecules, some of them have potent antimicrobial activity, including fosfomycin and fosmidomycin, and may be produced by microbes as antibiotics (Seto and Kuzuyama, 1999; Metcalf and van der Donk, 2009). The carbon-phosphorus bond is produced by coupled enzymes phosphoenolpyruvate phosphomutase (Ppm) and phosphonopyruvate decarboxylase (Ppd) to generate phosphonoacetaldehyde that is then converted to 2-aminoethylphosphonate (2-AEP) by AEP transaminase (Metcalf and van der Donk, 2009; Villarreal-Chiu et al., 2012). These are the first steps in the production of compounds containing the stable carbon-phosphorus bond.

**Figure 1:**
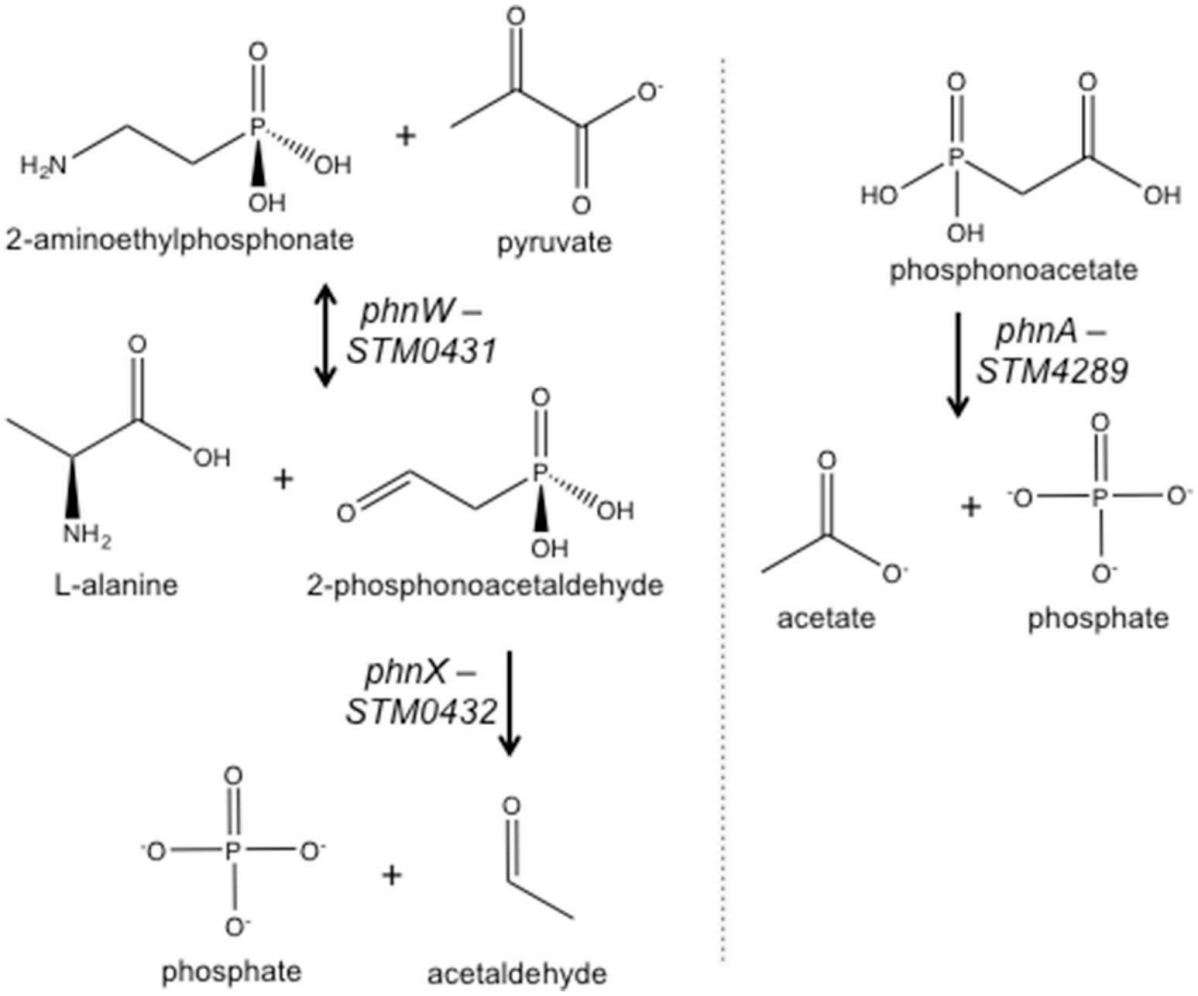
*****Salmonella*** enzymes with a putative role in phosphonate metabolism based on annotation**. See text for description. Not included is PhnO (STM4287), aminoalkylphosphonic acid N-acetyltransferase.

Although *Salmonella* lacks genes for the biosynthesis of phosphonates, it can utilize these compounds (Jiang et al., 1995; Errey and Blanchard, 2006). *Salmonella* has two loci annotated for degradation of phosphonates via the phosphonatase pathway, *phnABO* and *phnVUTSRWX* (Jiang et al., 1995). There are four genes annotated for phosphonate degradation, *phnW* (*STM0431*; 2-AEP-pyruvate aminotransferase), *phnX* (*STM0432*; phosphonatase), *phnA* (*STM4289*; phosphonoacetate hydrolase), and *phnO* (*STM4287*; aminoalkylphosphonic acid N-acetyltransferase) (Figure 1; Jiang et al., 1995; Errey and Blanchard, 2006). The *phnVUTSRWX* locus is under the control of the pho regulon and is activated during phosphate limitation (Jiang et al., 1995). *Escherichia coli* degrades phosphonates using the C-P lyase system encoded within a 14 gene operon (*phnCDEFGHIJKLMNO;* Metcalf and Wanner, 1993). Thus, the mechanism for phosphonate degradation seems to be different between *Salmonella* and its close relative.

We previously determined that a mutant lacking *STM3602* colonized ligated ileal loops in calves poorly (Elfenbein et al., 2013). In the calf model, *Salmonella* Typhimurium is inoculated into intestinal segments with undisturbed microflora and promotes a profound neutrophilic inflammatory response (Frost et al., 1997; Santos et al., 2002). Salmonellae must compete with the intestinal microbiota and withstand the host inflammatory response to survive in this model. Here we report that *STM3602* is necessary for colonization of the intestine of mice regardless of the composition of the microflora. A Δ*STM3602* mutant does not fully activate the TTSS-1 and has reduced epithelial cell invasion. However, this gene does not influence TTSS-2 expression and intracellular replication. The Δ*STM3602* mutant cannot grow if phosphonoacetic acid (PA) is provided as the sole source of phosphorus, but is capable of growth in 2-AEP. Finally, the gene encoding the known enzyme for PA breakdown, PhnA, does not appear to be under the control of *STM3602*, suggesting a different mechanism for utilization of PA. Our data suggest that *STM3602* may contribute to the regulation of various processes needed for STm to thrive in the intestine.

## Materials and methods

### Bacterial strains and plasmids

All bacterial strains are derivatives of ATCC 14028s and are listed in Table 1. Unless stated otherwise, bacteria were grown in Luria-Bertani (LB) broth or on LB agar supplemented with the following antibiotics as appropriate: kanamycin (50 mg/L), nalidixic acid (50 mg/L), carbenicillin (100 mg/L), streptomycin (100 mg/L), or chloramphenicol (20 mg/L). Mutants were constructed by a modification of the λ–red recombination technique and antibiotic cassettes were removed as described (Datsenko and Wanner, 2000; Santiviago et al., 2009). Chromosomal transcriptional fusions to *lacZY* were constructed as described previously (Ellermeier et al., 2002). All mutations were moved to a clean genetic background by bacteriophage P22-mediated transduction (Sternberg and Maurer, 1991).

**Table 1:** **Bacterial strains and plasmids**.

**Strain**	**Genotype**	**Reference or Source**
HA420	ATCC14028.s (Spontaneous Nal-R)	Bogomolnaya et al., 2008
HA964	14028 ΔSPI-1::*cm (*Cm-R*)*	Elfenbein et al., 2015
HA697	*ΔphoN*::*str* (Str-R)	Elfenbein et al., 2013
JE67	HA420 ΔphoN::str (Nal-R, Str-R)	Bogomolnaya et al., 2013
HA1473	HA420 Δ*STM3602*::kan (Kan-R, Nal-R)	Elfenbein et al., 2013
HA1474	HA420 Δ*STM3602*::kan + pWSK29::*STM3602* (Kan-R, Nal-R, Amp-R)	Elfenbein et al., 2013
JE60	HA420 Δ*STM3602*::kan + pWSK29 (Kan-R, Nal-R, Amp-R)	This study
JE346	HA420 Δ*STM3602*::frt (Nal-R)	This study
JE280	14028 Δ*phnA*::kan (Kan-R)	This study
JE347	HA420 Δ*phnA*::kan (Kan-R, Nal-R)	This study
HA1270	HA420 + pNN387 (Nal-R, Cm-R)	Zheng et al., 2013
HA1280	HA420 + pNN387::rpsMp (Nal-R, Cm-R)	Zheng et al., 2013
HA1315	HA420 + pNN387::prgHp (Nal-R, Cm-R)	Zheng et al., 2013
HA1319	HA420 + pNN387::ssaGp (Nal-R, Cm-R)	Zheng et al., 2013
HA1320	HA420 + pNN387::sseJp (Nal-R, Cm-R)	Zheng et al., 2013
JE248	HA1473 + pNN387::sseJp (Nal-R, Cm-R)	This study
JE249	HA1473 + pNN387::prgHp (Nal-R, Cm-R)	This study
JE250	HA1473 + pNN387::rpsMp (Nal-R, Cm-R)	This study
JE251	HA1473 + pNN387::ssaGp (Nal-R, Cm-R)	This study
JE255	HA1473 + pNN387 (Nal-R, Cm-R)	This study
JE169	HA1473 Δ*phoN*::str (Nal-R, Kan-R, Str-R)	This study
JE173	JE169 + pWSK29::*STM3602* (Nal-R, Kan-R, Str-R, Amp-R)	This study
JE175	JE169 + pWSK29 (Nal-R, Kan-R, Str-R, Amp-R)	This study
JE349	HA420 *phnA::lacZY* (Nal-R, Kan-R)	This study
JE351	JE346 *phnA::lacZY* (Nal-R, Kan-R)	This study
JE10.1	HA964 Δ*STM3602*::kan (Cm-R, Kan-R)	This study
**Plasmid**	**Description**	**Reference or Source**
pWSK29	Cloning vector; AmpR	Wang and Kushner, 1991
pWSK29::*STM3602*	pWSK29::*STM3602; AmpR*	Elfenbein et al., 2013
pCP20	flp recombinase; AmpR	Datsenko and Wanner, 2000
pCLF3.1	Template for Cm-R KO PCR product	Santiviago et al., 2009
pCLF4.1	Template for Kan-R KO PCR product	Santiviago et al., 2009
pKD46	Lambda-red recombinase; AmpR	Datsenko and Wanner, 2000
pKG136	pCE36; *lacZY* transcriptional fusion	Ellermeier et al., 2002

### Mouse infections

The Texas A&M University and University of California Davis Institutional Animal Care and Use Committees approved all animal experiments (approval numbers TAMU 2012-084 and 2011-167; UCD 19001). Mouse experiments were performed using 10–12 week old female C57BL/6J or CBA/J mice as indicated (Jackson Laboratories) as previously described (Barthel et al., 2003).

In the acute murine colitis model, C57BL/6J mice were administered 20 mg streptomycin in 75 μL sterile water by gavage. Twenty-four hours after treatment, mice were infected with ~10^8^ CFU of an equivalent mixture of WT and mutant bacteria in 100 μL volume by gavage. Feces were collected 24 h after infection. Mice were euthanized 96 h post-infection and organs harvested, homogenized, serially diluted, and plated on LB agar with appropriate antibiotics for enumeration of CFU.

In the chronic murine colitis model, CBA/J mice were administered 20 mg streptomycin in 75 μL water or 75 μL sterile water by gavage. Forty-eight hours after treatment, mice were infected as above. Feces were collected on the indicated days and mice euthanized 14 days post-infection. Competitive index was determined by comparing the ratio of WT to mutant bacteria in the tissue to that of the inoculum.

Germ-free Swiss Webster mice were bred and housed under germ-free conditions inside gnotobiotic isolators (Park Bioservices, LLC). Weekly cultures were grown to monitor the germ-free status of the mice. For experiments, male and female 6–8-week-old mice were transferred to a biosafety cabinet and maintained in sterile cages for the duration of the experiment. Mice were infected with ~10^8^ CFU of an equivalent mixture of WT and mutant bacteria in 100 μL volume by gavage. Mice were euthanized 24 h post-infection, cecal and colon contents were homogenized, serially diluted, and plated on LB agar with appropriate antibiotics for enumeration of CFU. The competitive index was determined by normalizing the ratio of WT to mutant bacteria in intestinal contents to that of the inoculum.

### Invasion assays

Cell lines were purchased from American Type Culture Collection (ATCC) and used within 15 passages. HeLa cells (human cervical adenocarcinoma epithelial, ATCC CCL-2) were grown as recommended by ATCC. HeLa cells were seeded in 24-well plates at 5 × 10^4^ cells/well ~24 h prior to infection.

Invasion assays were performed as previously described (Ibarra et al., 2010). Late-log phase cultures were prepared by inoculating 10 ml LB-Miller broth with 0.3 ml overnight culture. Flasks were grown at 37°C with aeration for 3 h. Bacteria were collected by centrifugation at 8,000 × g for 90 s, resuspended in an equal volume of Hanks' buffered saline solution (HBSS, Mediatech) and added directly to mammalian cells seeded in 24-well plates for 10 min. The multiplicity of infection was ~50:1 (bacteria:eukaryotic cell). Non-internalized bacteria were removed by aspiration, monolayers washed three times in HBSS and then incubated in growth media until 30 min post-infection. Gentamicin was added at 50 μg/ml from 30 to 90 min post-infection to kill extracellular bacteria and the media was replaced with media containing 10 μg/ml gentamicin from 90 min post infection. For enumeration of intracellular bacteria, monolayers were washed once in phosphate-buffered saline, solubilized in 0.2% (w/v) sodium deoxycholate and serial dilutions were plated on LB agar.

### ß-galactosidase assays

For induction of SPI-1 expression, bacterial cells bearing plasmid constructs were grown overnight in LB with appropriate antibiotics. Overnight cultures were diluted 1:100 and incubated at 37°C with agitation for 3 h. SPI-2 inducing media was used as described (5 mM KCl, 7.5 mM (NH_4_)_2_SO_4_, 0.5 mM K_2_SO_4_, 8 μM MgCl_2_, 337 μM KH_2_PO_4_, 80 mM MES, 0.3% (v/v) glycerol, 0.1% (v/v) casamino acids, pH 5.8) (Coombes et al., 2004). Cells were grown overnight in SPI-2 inducing media, diluted 1:50, and incubated for an additional 24 h to evaluate SPI-2 expression. To evaluate the expression of *phnA* in the presence of PA, overnight cultures were diluted 1:100 in LB with 40 mM MOPS at pH 6.8 with the indicated phosphorus source. Cultures were incubated at 37°C with agitation for 3 h.

ß-galactosidase activity was determined using standard methodology (Miller, 1972). Briefly, bacterial cells were pelleted by centrifugation and resuspended in Z-buffer (60 mM Na_2_HPO_4_, 40 mM NaH_2_PO_4_, 10 mM KCl, 1 mM MgSO_4_, 50 mM ß-mercaptoethanol). Culture density was determined by OD_600_. Bacteria were permeabilized with chloroform and 0.1% (w/v) SDS prior to addition of substrate (o-nitrophenyl-β-D-galactoside; ONPG 4 mg/mL). The reactions were performed at 28°C and were stopped with 1 M Na_2_CO_3_ for determination of OD_420_ and OD_550_. β-galactosidase activity (Miller units) was calculated using the following equation: 1000 × [OD_420_–(1.75 × OD_550_)] / [time × volume × OD_600_].

### Phosphonate growth

Modifications were made to a phosphorus-limited minimal medium (Neidhardt et al., 1974) to assess the ability of different bacterial strains to utilize different phosphorus sources. The final media composition (MMMM) was as follows: 20 mM NH_4_Cl, 2.5 mM Na_2_SO_4_, 80 mM NaCl, 0.35 mM CaCl_2_, 20 mM KCl, 40 mM MOPS, 1 mM MgSO_4_, 0.01 mM FeSO_4_, 0.2% (w/v) glucose, pH 6.8. Phosphorus sources were Na_2_HPO_4_ (Pi, Sigma), 2-aminoethylphosphonate (2-AEP, Sigma), and phosphonoacetic acid (PA, Sigma) added at the concentrations indicated. Bacterial strains were grown overnight at 37°C with agitation in MMMM with Na_2_HPO_4_ at the indicated concentration. Overnight cultures were pelleted and pellets were resuspended in an equal volume MMMM supplemented with the indicated phosphorous sources. After re-centrifugation and resuspension in the starting volume or the original culture, bacteria were diluted 1:100 into fresh MMMM with indicated phosphorus source. Aliquots were removed, serially diluted, and plated to determine CFU.

### Data analysis

All data were log transformed prior to analysis. Statistical significance was determined using Student's *t*-test or analysis of variance with significance set at *P* < 0.05.

## Results

### *STM3602* in intestinal colonization

**Figure 2:**
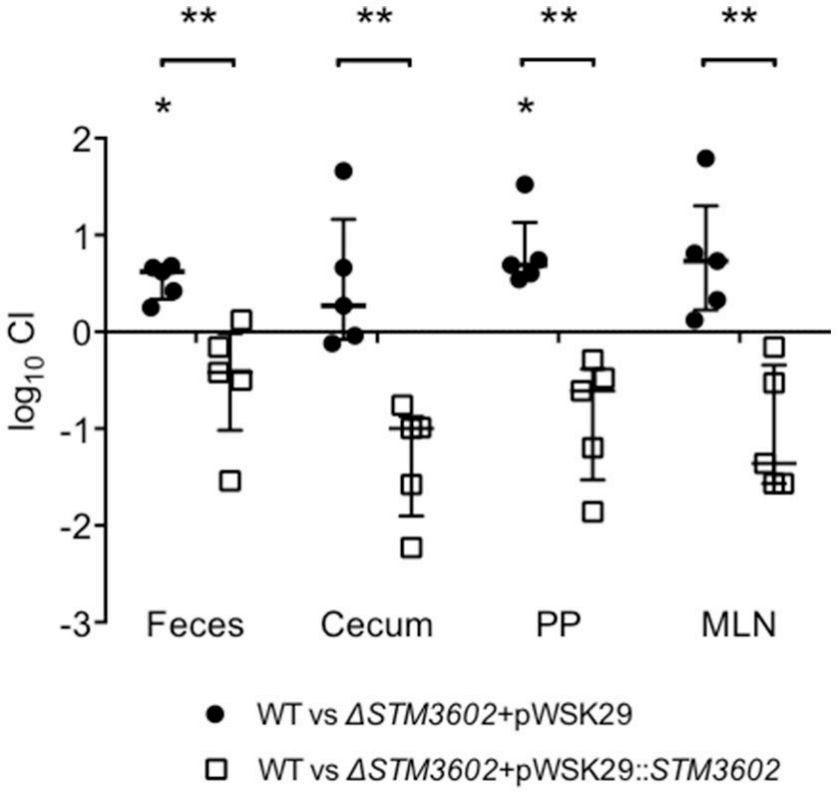
**During acute colitis, the Δ***STM3602*** mutant colonizes the murine Peyer's patches and mesenteric lymph nodes poorly**. Ten C57BL/6 mice were treated with streptomycin (20 mg) then infected with ~10^8^ CFU of an equivalent mixture of WT (JE67) and Δ*STM3602* mutant bearing an empty plasmid (JE175) or complementing plasmid (JE173) 24-h after antibiotic treatment. Feces (F) were collected 24 h after infection and mice were euthanized 96-h post-infection for collection of Peyer's patches (PP), mesenteric lymph nodes (MLN), and cecum (C). The competitive index (CI) was determined by comparing the ratio of WT to mutant in the tissue to that of the inoculum. Each data point represents a single mouse with median and interquartile range indicated by horizontal bars. Statistical significance was determined by Student's *t*-test, and is indicated by an asterisk (^*^), and statistically significant differences between groups is indicated by two asterisks (^**^) with *P* < 0.05.

*STM3602* is necessary for STm to colonize the intestine of calves in ligated ileal loops (Elfenbein et al., 2013). We used the murine colitis model to further dissect the function of this gene during infection (Barthel et al., 2003). In this model, mice are treated with high doses of streptomycin prior to infection promoting STm colonization and development of a neutrophilic inflammatory response. We found that the Δ*STM3602* mutant is shed in lower numbers than the wild type organism in the feces by 24 h post-infection, and colonizes Peyer's patches and mesenteric lymph nodes at lower levels than the wild type organism at 4 days post-infection (Figure 2). This colonization defect was reversed by complementation *in trans*. Colonization of the cecum by the Δ*STM3602* mutant was highly variable in different mice making it difficult to determine with statistical confidence whether or not mutants lacking *STM3602* have a colonization defect in the cecum. Regardless, our results are consistent with previous data suggesting a requirement of *STM3602* in colonization of the intestine in the presence of a profound neutrophilic inflammatory response.

### *STM3602* and SPI-1 regulation

Salmonellae possess two type-3 secretion systems, encoded by *Salmonella* pathogenicity islands 1 and 2 (SPI-1 and SPI-2). The TTSS-1 and its associated effectors are essential for invasion of non-phagocytic epithelial cells and penetration of the intestinal epithelium (Zhang et al., 2002; Raffatellu et al., 2005). The TTSS-2 is needed for maintenance of the *Salmonella*-containing vacuole and intracellular replication (Figueira and Holden, 2012). We hypothesized that the intestinal colonization defect of Δ*STM3602* mutants could be due to reduced expression of TTSS-1.

**Figure 3:**
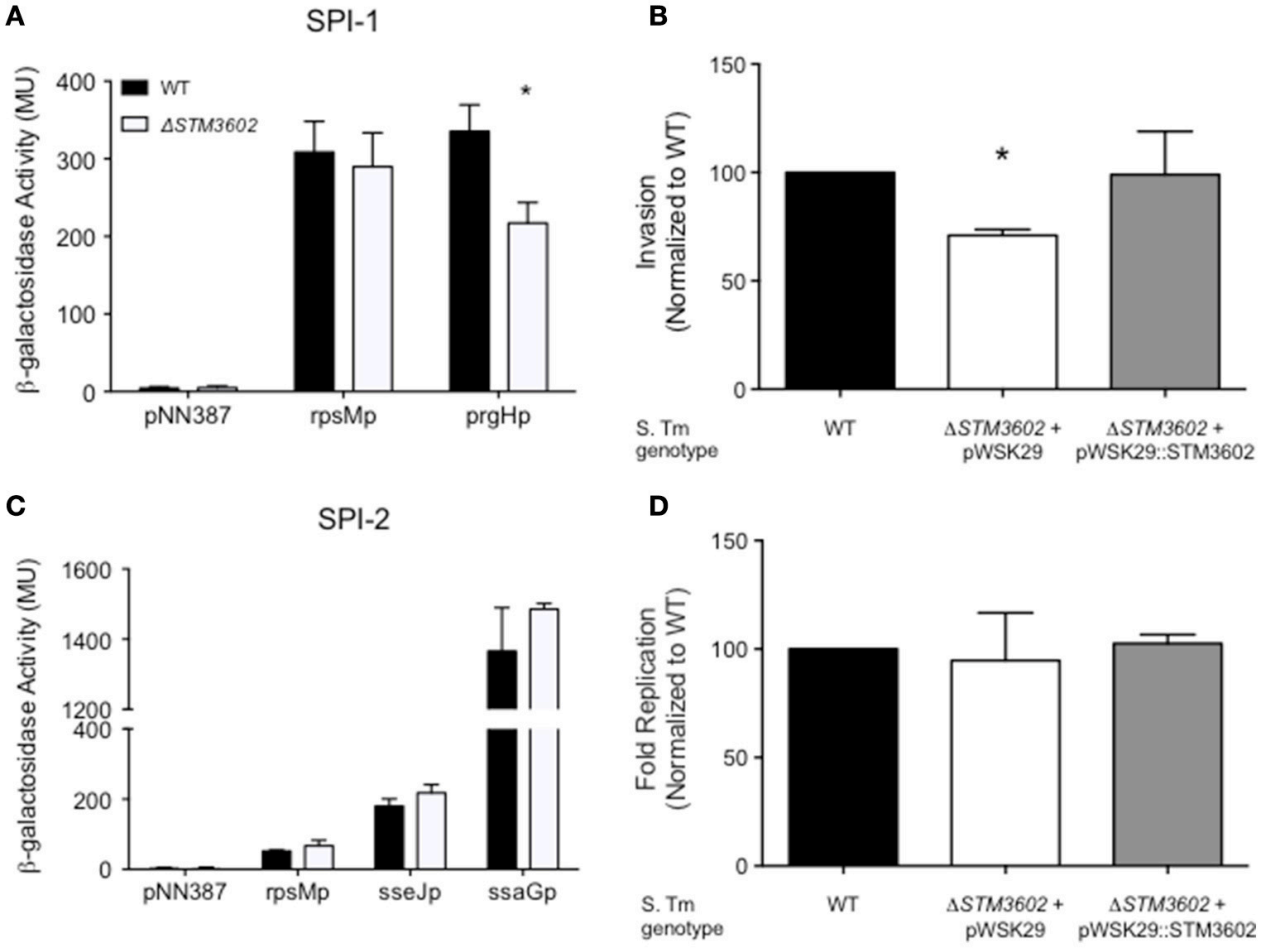
**The Δ***STM3602*** mutant has reduced invasion efficiency into cultured epithelial cells due to poor activation of SPI-1. (A)** Activation of a terminal SPI-1 promoter (*prgH*p-*lacZY*) in SPI-1 inducing conditions as determined by ß-galactosidase activity. **(B)** Invasion efficiency of Δ*STM3602* (JE60) and complemented Δ*STM3602* (HA1474) mutants into HeLa epithelial cell monolayers normalized to the efficiency of the WT (HA420) at 1 h post-infection. **(C)** Activation of two SPI-2 terminal promoters (*sseJ*p-*lacZY* and *ssaG*p-*lacZY*) in SPI-2-inducing conditions as determined by ß-galactosidase activity. **(D)** Fold-replication of Δ*STM3602* and complemented Δ*STM3602* mutants in HeLa cells; 7 h post-infection/1 h post-infection normalized to WT fold-replication. Bars represent the mean ± *SD*. Assays were performed on three separate occasions. Statistical significance (^*^) was determined by Student's *t*-test with *P* < 0.05.

We used plasmids containing *lacZY* under the control of promoters of genes on SPI-1 or SPI-2 to determine whether deletion of *STM3602* resulted in an effect on activation of these promoters (Zheng et al., 2013). Using a plasmid containing *lacZY* under the control of the *prgH* promoter (a TTSS-1 structural gene), we found that the *prgH* promoter is activated less efficiently in SPI-1 inducing conditions in bacteria lacking *STM3602* than in the WT (Figure 3A). Consistent with this result, deletion of *STM3602* also results in a similar reduction in invasion in cultured epithelial cells (Figure 3B). Conversely, the Δ*STM3602* mutant is capable of activation of TTSS-2 apparatus (*ssaG*) and effector promoters (*sseJ*; Figure 3C) as well as survival within cultured epithelial cells to the same level as the wild type (Figure 3D). These results suggest that *STM3602* plays a role in the complex regulatory network of the TTSS-1.

### Growth in phosphonoacetate as sole phosphorus source

**Figure 4:**
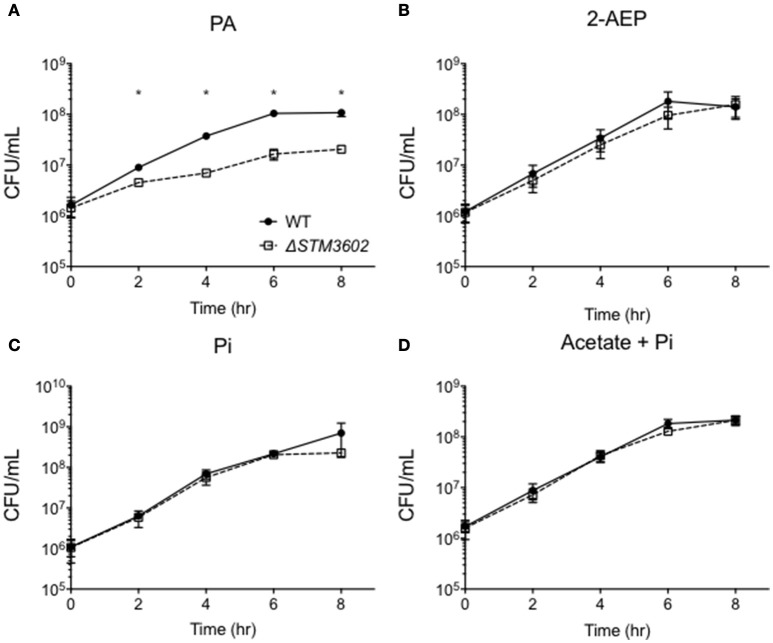
*****STM3602*** is required for adequate growth with phosphonoacetic acid as a sole phosphorus source. (A–D)** Growth curve of WT (HA420) and Δ*STM3602* (HA1473) in a minimal medium supplemented with 5 mM PA **(A)**, 2-AEP **(B)**, Pi **(C)** and 5 mM sodium acetate with 5 mM Pi **(D)**. Bacteria were grown overnight in MMMM with 5 mM Pi then diluted 1:100 into media with the indicated phosphorus and/or carbon additions. CFU were determined every 2 h on three independent occasions. Data points represent mean ± SEM. CFU data were log transformed and statistical significance determined by ANOVA. Asterisk (^*^) indicates significant difference between Δ*STM3602* and WT with *P* < 0.05.

*STM3602* belongs to the GntR family of transcriptional regulators and is a part of the PhnR clade (Marchler-Bauer et al., 2015). This gene shares 29.2% sequence similarity and 45.1% amino acid identity with PhnR (STM0430; Sievers et al., 2011). We hypothesized that STM3602 might regulate one or both of the phosphonate utilization loci in STm. We characterized the survival and growth of the Δ*STM3602* mutant in media containing either 2-AEP or PA as the sole phosphorus source. When strains were grown in the presence of 5 mM PA, the ΔSTM3602 mutant replicated poorly (Figure 4A). However, this mutant grows normally in 5 mM 2-AEP or Pi (Figures 4B,C) as sole phosphorus sources. PA is degraded to acetate and inorganic phosphate (Figure 1). One possible explanation for the phenotype of the Δ*STM3602* mutant in PA-containing medium is that this mutant may be unable to utilize or excrete acetate. However, the Δ*STM3602* mutant exhibits similar growth kinetics to WT in the presence of both 5 mM acetate and 5 mM Pi (Figure 4D) suggesting a mechanism specific to use of PA. Overall, these data suggest a role for *STM3602* in phosphonoacetate utilization.

**Figure 5:**
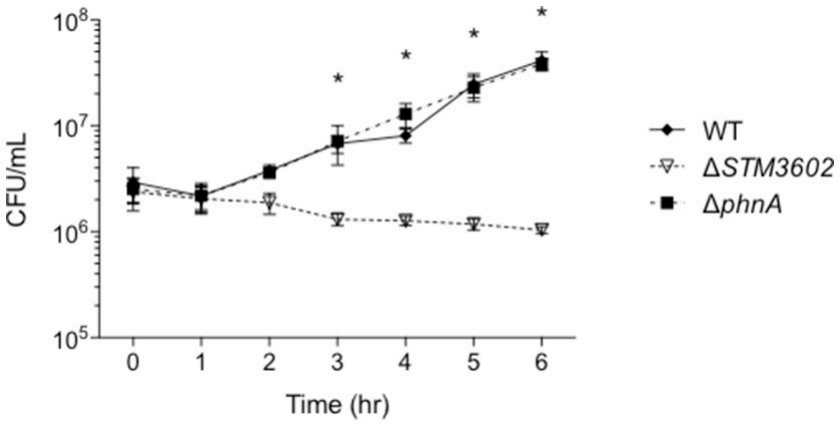
**Growth in PA as a sole phosphorous source requires ***STM3602*** but not ***phnA*****. Bacterial strains were grown overnight in 0.5 mM Pi and diluted 1:100 into media with 5 mM PA. CFU were determined hourly. CFU data were log transformed and statistical significance determined by ANOVA. (^*^) indicates significant difference between Δ*STM3602* and WT with *P* < 0.05.

Next, we determined the growth kinetics of the Δ*STM3602* mutant in media with 5 mM PA after phosphorus deprivation. When bacteria were grown in phosphorus-limiting conditions (0.5 mM Pi) and transferred to media containing 10 times more phosphorus in the form of PA, the Δ*STM3602* mutant lost viability over the course of the 6-h experiment (Figure 5). A mutant deleted for *phnA*, a gene that encodes the phosphonoacetate hydrolase, had growth kinetics similar to the WT in these conditions (Figure 5). These results suggest that *STM3602* is needed for activation of pathways necessary for growth in the presence of PA and that there is a pathway for metabolism of this compound that operates independently of *phnA*.

### Expression of *phnA*

**Figure 6:**
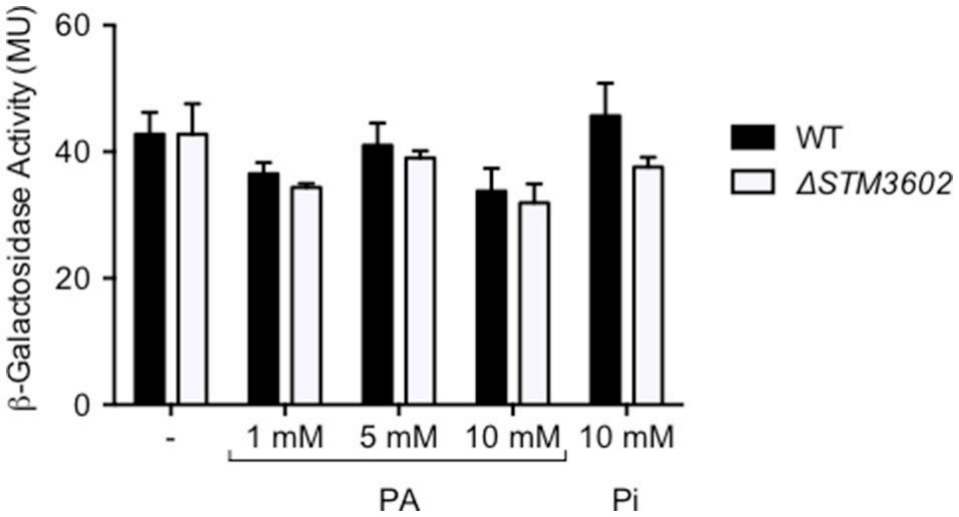
**Deletion of ***STM3602*** does not affect ***phnA*** expression**. The expression of *phnA::lacZY* was determined in log phase cultures in rich medium by measuring ß-galactosidase activity in both the WT (black bars, JE349) and Δ*STM3602* mutant (white bars, JE351) backgrounds. Bars represent the mean ± SEM of 3 independent experiments.

The Δ*STM3602* mutant grows poorly in the presence of PA. We hypothesized that *STM3602* regulates the *phnABO* operon because the annotation of *phnA* suggests that PhnA may degrade PA. In order to test this hypothesis, we generated a mutant strain bearing a *lacZY* fusion to the first 10 amino acids of *phnA* to monitor transcription from the native *phnA* promoter. Using this construct, we found that in rich media the expression of the *phnA* promoter was not affected by the addition of PA at varying concentrations (Figure 6). In addition, deletion of *STM3602* did not affect the expression of *phnA* in these conditions during log phase growth (Figure 6), a condition where *STM3602* is expressed (Kroger et al., 2013). These data suggest that STM3602 does not regulate *phnA* in rich medium in the presence or absence of PA.

### Impact of microbiota on Δ*STM3602* intestinal colonization

The first phosphonate to be discovered, 2-AEP is present in both ruminal and duodenal contents of sheep (Ankrah et al., 1989). This compound is associated with ruminal protozoal and bacterial populations and is less abundant in defaunated animals compared with those with a normal microbial composition. Phosphonoacetic acid is one product of 2-AEP metabolism mediated by the enzyme PhnY, phosphonoacetaldehyde oxidase (Agarwal et al., 2014). Although salmonellae lack a gene with this annotated function, phyla found in the murine intestinal microflora including Cyanobacteria, Proteobacteria and Firmicutes encode genes to produce PA from 2-AEP (Stecher et al., 2007; Martinez et al., 2010; Villarreal-Chiu et al., 2012).

**Figure 7:**
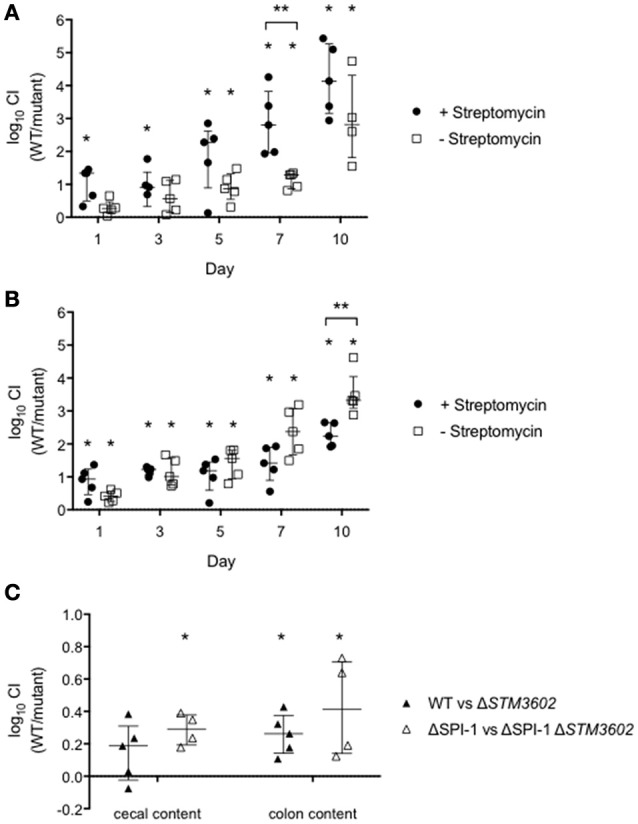
**The colonization defect of the Δ***STM3602*** mutant is independent of the host inflammatory response and microbial composition. (A,B)** Ten CBA/J mice were treated with streptomycin (20 mg; closed circles) or an equivalent volume of sterile water (open squares) and infected 48 h later with ~10^8^ CFU of an equivalent mixture of **(A)** WT (HA420) and Δ*STM3602* mutant (HA1473) or **(B)** ΔSPI-1 (HA964) and ΔSPI-1 Δ*STM3602* (JE10.1) mutant by gavage. Feces were collected on the indicated days. **(C)** Nine germ free Swiss Webster mice were infected with 10^8^ CFU of an equivalent mixture of WT and Δ*STM3602* mutant (closed circles) or ΔSPI-1 and ΔSPI-1 Δ*STM3602* mutant (open squares) by gavage. Animals were sacrificed 1 day post-infection and bacterial numbers enumerated from cecal and colon contents. Significant difference in CI (WT/mutant) is indicated by an asterisk (^*^) and difference between groups is indicated by two asterisks (^**^) with *P* < 0.05. Analyses for statistical significance determined as in Figure 2.

We hypothesized that the colonization defect of the Δ*STM3602* mutant might correlate with microbial colonization of the host intestine. We used the chronic carriage mouse model to determine the longer-term kinetics of the colonization defect of this mutant. In streptomycin pre-treated mice (altered microbiota) in competitive infection with the WT, the Δ*STM3602* mutant failed to colonize the intestine beginning very early at 1-day post-infection. In animals with an intact microbiota (no streptomycin pretreatment) the colonization defect of the Δ*STM3602* mutant took longer to develop becoming apparent at 5 days post infection (Figure 7A). Thus, the colonization defect of the Δ*STM3602* mutant occurs much earlier in animals with disrupted microbiota than with intact microbiota.

One possible explanation for the reduced ability of the Δ*STM3602* mutant to colonize the murine intestine is due to reduced expression of the TTSS-1 (Figures 3A,B). To rule out this possibility, we performed a competitive infection experiment in a ΔSPI-1 genetic background (ΔSPI-1 mutant vs. ΔSPI-1Δ*STM3602* double mutant). In these experiments, the double ΔSPI-1Δ*STM3602* mutant colonizes the intestine poorly on the first day post-infection in animals regardless of whether the intestinal microflora were intact or disrupted (Figure 7B). The phenotype of the Δ*STM3602* mutant was significantly stronger 10 days post-infection in the animals that began with disrupted microflora. These data suggest that the observed colonization defects are unlikely to be due solely to poor activity of the TTSS-1 but more likely a result of inability to compete in the intestine.

Finally, we evaluated the fitness of the Δ*STM3602* mutant using competitive infections in germ-free mice to eliminate any effects of microbiota on colonization efficiency of the Δ*STM3602* mutant. In the germ free murine model, the Δ*STM3602* mutant exhibited a small but significant colonization defect in the cecal contents in the WT and ΔSPI-1 genetic backgrounds (Figure 7C) but only a significant colonization defect in the colon contents in the ΔSPI-1 genetic background. Taken together, these data suggest that STM3602 is required for colonization of the murine intestine regardless of the host inflammatory response or the microbial composition of the intestine.

## Discussion

We report that the putative GntR family regulator STM3602 is necessary for colonization of the murine intestine, consistent with the reduced ability of this mutant to colonize the bovine intestine (Elfenbein et al., 2013). Two possible mechanisms for these colonization defects are: (1) a role for STM3602 in the activation of the TTSS-1 and/or (2) a role in use of the microbial-derived product, phosphonoacetic acid. Our data are consistent with a regulatory role of STM3602 in modulating STm virulence and use in the intestine of the infected animal.

2-AEP was the first phosphonate discovered (Horiguchi and Kandatsu, 1959). This compound is found in low amounts in feed and is associated with bacterial and protozoan populations within the intestine (Ankrah et al., 1989). 2-AEP is also found in mammalian tissues likely from assimilation from microbial and feed sources because mammals lack the enzymes to produce such molecules (Shimizu et al., 1965; Alhadeff and Daves, 1971; Metcalf and van der Donk, 2009). Numerous marine bacterial phyla contain genes for the biosynthesis and degradation of phosphonates (Villarreal-Chiu et al., 2012). However, the production of phosphonates by microbes is not restricted to the marine biosphere. The leading bacterial phyla containing phosphonate biosynthetic genes from mammalian and bird microbiome metagenomes are Firmicutes, Proteobacteria, and Bacteroidetes (Yu et al., 2013). These phyla are abundant in the murine cecum, although their relative abundance is substantially altered following streptomycin treatment (Stecher et al., 2007).

A mutant in Δ*STM3602* has both a colonization defect in the intestine of mammals and fails to utilize phosphonoacetic acid as a sole phosphorus source. The relationship between these phenotypes remains unclear. We and others have previously shown that a Δ*STM3602* mutant is defective for intestinal colonization of calves and pigs (Carnell et al., 2007; Elfenbein et al., 2013). Here we found that the Δ*STM3602* mutant also poorly colonizes the intestine of mice. This colonization defect persists regardless of the character of intestinal microbiota, and is also present both in the presence and absence of TTSS-1 induced inflammation. Thus, the host may act as a source of phosphonates, having absorbed them from the microflora or feed sources and incorporated them into phosphonolipids (Shimizu et al., 1965; Alhadeff and Daves, 1971). Further studies evaluating the phosphonate content of intestinal fluid and tissue in animals will be very informative.

The only gene in the STm genome with putative function to catalyze the conversion of phosphonoacetate to inorganic phosphate and acetate is *phnA*. No studies have evaluated the regulation of *phnABO*. Our data suggest that *phnA* is expressed at low levels in rich medium and that the expression of *phnA* is not induced in the presence of PA nor is it required for growth with PA as a sole phosphorus source. Our data also suggest that the expression of *phnA* is not affected by deletion of *STM3602*. We measured *phnA* expression during log phase growth, a time when *STM3602* is expressed (Kroger et al., 2013). The phosphorus content of LB broth is undefined, and it is possible that the expression profile of *phnA* would differ in the defined media in the presence of different phosphorus sources. The Δ*STM3602* mutant is unable to utilize PA as a sole phosphorus source but the Δ*phnA* mutant can. One reason may be that the Δ*STM3602* mutant fails to activate a transporter for PA. While this would explain the lack of growth in the Δ*STM3602* mutant, it does not explain the finding that the Δ*phnA* mutant grows with WT efficiency with PA as a sole phosphorous source. This finding suggests that there may be an additional mechanism for catalysis of PA encoded elsewhere in the genome that is likely under the regulatory control of STM3602.

STM3602 is a member of the GntR family of regulators, characterized by N-terminal DNA-binding domains and variable C-terminal small ligand binding domains (Haydon and Guest, 1991; Gorelik et al., 2006). The crystal structure of PhnF of both *E. coli* and *M. smegmatis*, a regulator of the C-P lyase pathway for phosphonate catalysis and member of the GntR family of regulators has been solved and a small molecule ligand has been modeled into a binding site although the actual ligand remains unknown (Gorelik et al., 2006; Gebhard et al., 2014). STM3602 shares a conserved domain with PhnF, but has only 23.4% amino acid identity across the entire protein. *Salmonella* lacks the gene *phnF* but has a putative regulator of phosphonate utilization, *phnR* (*STM0430*). STM3602 has 45.1% amino acid identity with PhnR (Sievers et al. 2011). The GC content of *STM3602* and *phnR* differ substantially (49 and 59%, respectively), consistent with recent acquisition of *STM3602* likely with a mechanism independent of the function of *phnR*.

Non-typhoidal *Salmonella* use the TTSS-1 to invade normally non-phagocytic intestinal epithelial cells and induce a strong neutrophilic inflammatory response (Tsolis et al., 1999; Zhang et al., 2002; Raffatellu et al., 2005). The central importance of this pathogenicity island is illustrated by the fact that salmonellae lacking a functional TTSS-1 are avirulent in calf, pig, and mouse models of infection (Tsolis et al., 1999; Zhang et al., 2002; Barthel et al., 2003; Coombes et al., 2005). The regulatory network controlling the expression of the TTSS-1 is intricate. Transcriptional regulation is carefully controlled by regulatory proteins encoded both within SPI-1 and located elsewhere on the chromosome (Ong et al., 2010). The master regulator of the TTSS-1, *hilA*, is encoded within SPI-1 and integrates regulatory input from numerous sources to activate operons necessary for the production of protein components of the TTSS-1 and its associated effectors (reviewed in Ellermeier and Slauch, 2007). We have shown a minor role for STM3602 in the regulation of SPI-1 and invasion of tissue cultured epithelial cells. However, a Δ*STM3602* ΔSPI-1 double mutant was more severely compromised for colonization of the murine intestine than a mutant lacking only SPI-1 (Figure 7). This finding suggests that STM3602 plays a role in intestinal colonization that is not fully explained by inadequate activation of SPI-1. However, the role of STM3602 in regulation of SPI-1 remains an interesting area of further study to contribute to the breadth of knowledge on this essential virulence mechanism.

The genes neighboring *STM3602* have recently been described as important for utilization of fructose-asparagine during colonization of the mouse intestine (Ali et al., 2014; Sabag-Daigle et al., 2016). These genes (*STM3602-STM3598*; *fraRBDAE*) share a similar GC content (47.7–51.3%) so were likely acquired together. However, it is unclear whether these genes are co-transcribed from the same promoter or whether they function in the same genetic pathway. The colonization defect of the Δ*STM3601* mutant compared with its isogenic WT is substantially greater than that of the Δ*STM3602* mutant after 1 day infection in germ-free animals (Figure 7C), suggesting that these genes may not operate in the same genetic pathway (Ali et al., 2014). Further studies are needed to determine whether these genes belong to an operon or have shared functions during infection.

We have confirmed that STM3602 is needed for colonization of the mammalian intestine. We report that a deletion mutant lacking *STM3602* grows poorly with PA as a sole phosphorus source and that STM3602 has no effect on the expression of *phnA*, the enzyme annotated for utilization of this compound. Finally, we show that STM3602 plays a minor role in the regulation of the TTSS-1. Phosphonates are compounds that are produced by microorganisms and have been identified in the intestine of mammals as a result of microbial colonization. Further, studies evaluating the role of STM3602 in co-culture with microorganisms known to produce diverse phosphonates will elucidate the full complement of compounds to which this regulator responds. In addition, definition of the STM3602 regulon may elucidate novel mechanisms for phosphonate transport or utilization in the large complement of genes of unknown function scattered within the genome of STm.

## Author contributions

JE and HA designed the experiments, analyzed the data, and drafted the manuscript. JE, LK, AS, and FF performed the experiments. JE, LK, FF, AB, and HA edited the manuscript.

## Funding

This work was supported by NIH grant R01 AI03646 and to HA. JE was supported by Office of Research Infrastructure Programs/OD NIH grant 8T32OD011083. LK was supported by start-up funds from the Paul G. Allen School for Global Animal Health. Work in the AB laboratory is supported by NIH AI096528.

### Conflict of interest statement

The authors declare that the research was conducted in the absence of any commercial or financial relationships that could be construed as a potential conflict of interest.
